# Three-Dimensional Assignment of the Structures of Atomic Clusters: an Example of Au_8_M (M=Si, Ge, Sn) Anion Clusters

**DOI:** 10.1038/srep17738

**Published:** 2015-12-03

**Authors:** Yi-Rong Liu, Teng Huang, Yan-Bo Gai, Yang Zhang, Ya-Juan Feng, Wei Huang

**Affiliations:** 1 Laboratory of Atmospheric Physico-Chemistry, Anhui Institute of Optics & Fine Mechanics, Chinese Academy of Sciences, Hefei, Anhui 230031, China; 2 School of Environmental Science & Optoelectronic Technology, University of Science and Technology of China, Hefei, Anhui 230026, China

## Abstract

Identification of different isomer structures of atomic and molecular clusters has long been a challenging task in the field of cluster science. Here we present a three-dimensional (3D) assignment method, combining the energy (1D) and simulated (2D) spectra to assure the assignment of the global minimum structure. This method is more accurate and convenient than traditional methods, which only consider the total energy and first vertical detachment energies (VDEs) of anion clusters. There are two prerequisites when the 3D assignment method is ultilized. First, a reliable global minimum search algorithm is necessary to explore enough valleys on the potential energy surface. Second, trustworthy simulated spectra are necessary, that is to say, spectra that are in quantitative agreement. In this paper, we demonstrate the validity of the 3D assignment method using Au_8_M^−^ (M = Si, Ge, Sn) systems. Results from this study indicate that the global minimum structures of Au_8_Ge^−^ and Au_8_Sn^−^ clusters are different from those described in previous studies.

Nanoclusters, including small groups of atoms or molecules, possess an intermediate size range between single atoms and condensed matter. Owing to the unique properties of the clusters in designing new types of nanofunctional materials, many efforts have been devoted to demonstrating that the properties of the clusters depend on their structure and composition^1^,^2^. Once low-lying structures are found, their properties can be calculated and compared with measured values to make precise structural assignments. Therefore, it is very important to determine the exact structure and composition of these nanoclusters^3^,^4^.

In the past two decades, various techniques have been used to investigate the structures of nanoclusters, such as photoelectron spectroscopy^5^,^6^,^7^,^8^,^9^,^10^, ion mobility^11^,^12^, infrared multiphoton dissociation spectroscopy^13^,^14^, electron diffraction^15^, X-ray diffraction^16^, coulomb explosion^17^,^18^,^19^, and trapped ion electron diffraction^20^. All of these techniques are quite powerful for obtaining structural information when combined with optimization algorithms and density functional theory (DFT) calculations. So far, the different types of algorithms have been developed to search the low-energy structure on the complicated potential energy surface (PES), such as genetic algorithms (GA)^21^, simulated annealing (SA)^22^, and basin hopping (BH)^23^,^24^,^25^,^26^. These experimental techniques and optimization algorithms are very useful for finding the low-energy structure on PES. However, the assignment of cluster structures remain to be difficult, specifically identifying the structural and energetic close isomers^27^. Therefore, determining the different isomers and confirming which structure is the global minimal structure in low-lying structures is still a fundamental problem in atomic clusters.

The conventional method used to distinguish the global minimum from other isomers is to calculate their relative energies and compare the calculated vertical detachment energies (VDEs) with the experimental value^28^. This methodology is not very effective at recognizing the lowest energy structure and the energetically close isomers^27^ because the relative energy calculations depend on the method and basis set. The different method and basis set can lead to a change of energy order. On the other hand, normally the vertical detachment energy differences of different isomers, especially for the low energy isomers, are very small. Therefore, it is difficult to distinguish the lowest energy structure from different isomers only by the relative energy and VDE values. To more reliably identify the global minimum from all of the isomers, we present a three-dimensional (3D), including energy (1D) and density of state (DOS) (2D), assignment method for effectively analyzing different low-energy structures of anion clusters. The simulated DOS spectra are based on a generalized Koopmann’s theorem^29^. It can effectively simulate the photoelectron spectra obtained by experiment to make the structure assignment. We used Au_8_M^−^ (M = Si, Ge, Sn) as an example system to illustrate the validity of our method. We then compared the DOS spectra for the isomers of Au_8_M^−^ (M = Si, Ge, Sn) systems with previous studies, thereby providing considerable credence for the identified isomers of these clusters.

## Results

**Figure 1:**
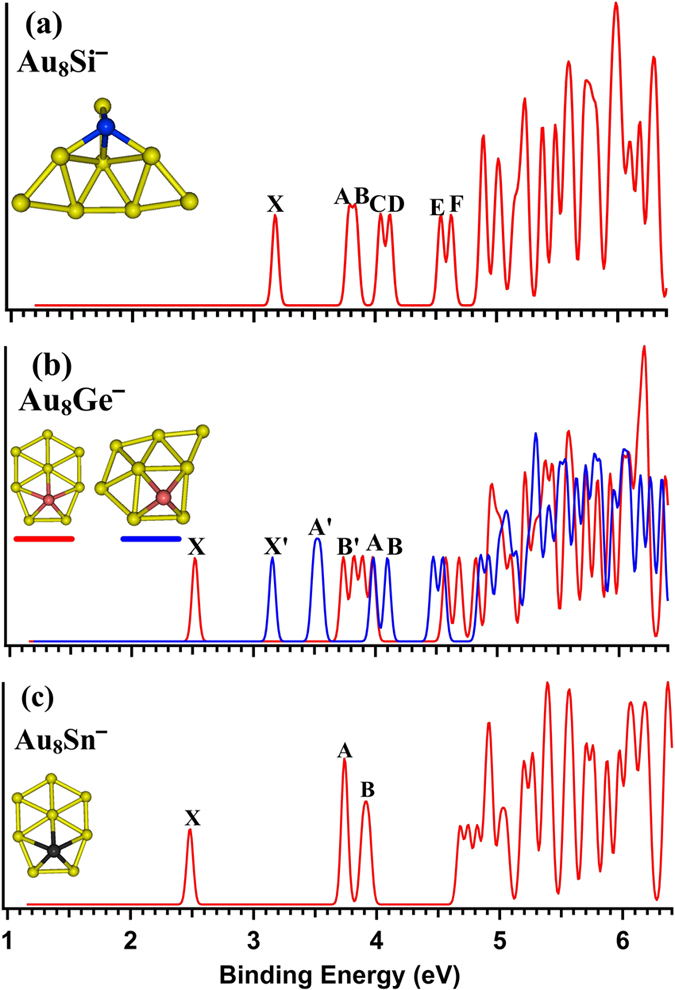
The simulated photoelectron spectra of Au_8_M^−^ (M = Si, Ge, Sn). The insets show the corresponding structures. The dopant atoms are shown in color (Si in blue, Ge in red, and Sn in black).

**Figure 2:**
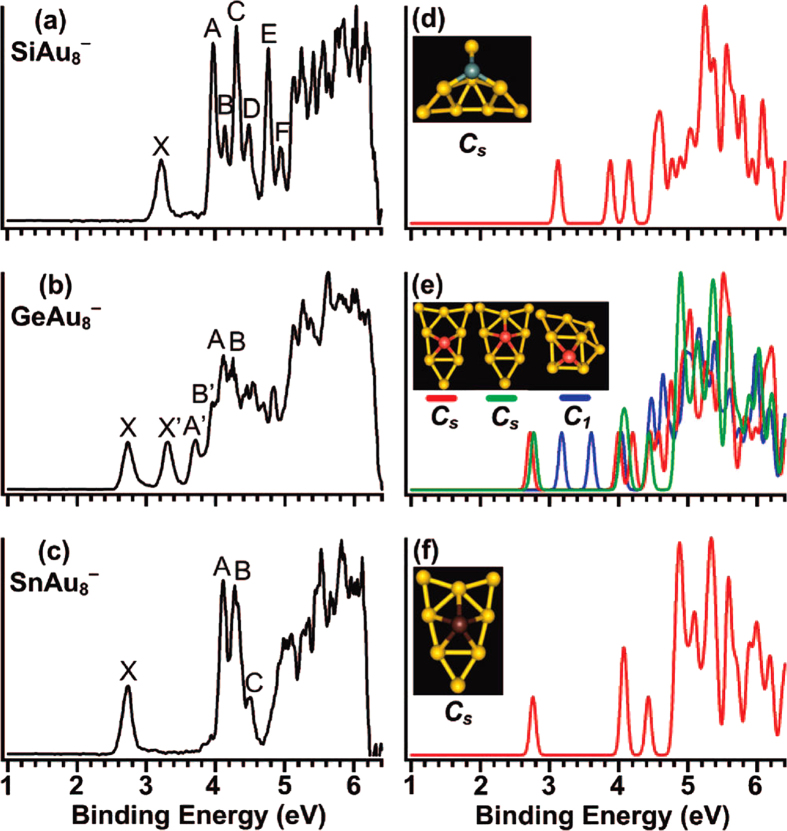
The experimental (left) and simulated (right) photoelectron spectra of Au_8_M^−^ (M = Si, Ge, Sn). with the permission ref. 31. Copyright 2009 American Chemical Society.

To determine the global minima of the Au_8_M^−^ (M = Si, Ge, Sn) systems, we searched more than 200 isomeric forms using the BH method^23^ Table 1 lists the relative energies of the top 5 isomers of Au_8_M^−^ (M = Si, Ge, Sn) systems at several levels of theory (see the table titles). All the coordinates of the top 5 low energy structures of the Au_8_M^−^ (M = Si, Ge, Sn) systems can be found in the Supporting Information. The first VDEs of each species are calculated at the PBE0/CRENBL (SO) level of theory (using the NWChem software package^30^) and compared with the experimental values obtained by Wang *et al*.^31^ in Table 1. The simulated spectra of the primary structures of the Au_8_M^−^ (M = Si, Ge, Sn) systems are depicted in Fig. 1. The top 5 lowest-energy structures of the Au_8_M^−^ (M = Si, Ge, Sn) systems, together with their simulated PES spectra, are depicted in Figure S1 in the Supporting Information. The experimental spectra of the Au_8_M^−^ (M = Si, Ge, Sn) systems are showed in Fig. 2a~c.

**Figure 3:**
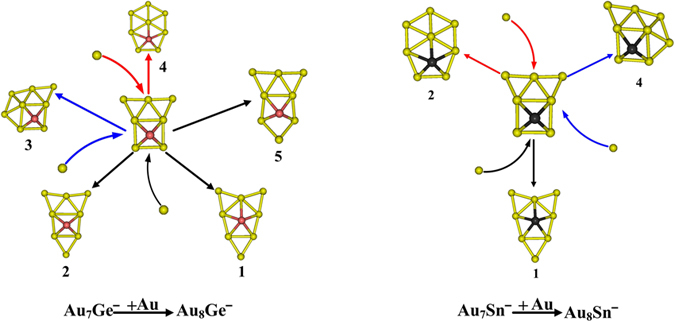
The structure evolution of the clusters from Au_7_M^−^ to Au_8_M^−^ (M = Ge, Sn). The structure of the Au_7_M^−^ (M = Ge, Sn) cluster was obtained from the literature^31^. The dopant atoms are shown in color (Ge in red, and Sn in black). The numbers are the same as in Table 1.

Table 1 shows that the results calculated from different theoretical levels lead to different energy values. Therefore, it is difficult for us to distinguish which isomer is the global minimum by their total energy and first VDEs. To accurately obtain the lowest energy structure, we compared their total energy and DOS spectra with the experimental results. The simulated spectrum of isomer 1 for the Au_8_Si^−^ cluster agrees well with the experimental data (Fig. 2a) obtained by Wang, *et al*.^31^ and should be the lowest-energy structure by our calculation. Due to the spin-orbit effects included for the Au atom, our simulated spectrum is better than previous studies (Fig. 2d)^31^, which had not considered the spin-orbit effects for the Au atom. The experimental spectra (Fig. 2b,c) of Au_8_Ge^−^ and Au_8_Sn^−^ clusters are very similar to the literature^31^, which suggests that their primary structures should be similar with each other. Based on the comparison of the total energy and DOS spectra of isomers 1 and 2 for the Au_8_Sn^−^ cluster, we confirmed that the primary structure of Au_8_Sn^−^ should be isomer 2. The simulated spectrum of isomer 2 agrees well with the experimental spectra (Fig. 2c) and suggests that isomer 2 is more likely to exist under real conditions. Comparing experimental and theoretical spectra for the Au_8_Ge^−^ cluster^31^, the simulated spectrum of isomer 4 (Figure. S1i in the Supporting Information) together with isomer 3 (Figure. S1h in the Supporting Information) has good agreement with the experimental spectrum (Fig. 2b). However, isomer 4 has a relatively higher energy than the other structures at several different theoretical levels (Table 1). Due to the similar experimental spectra of the Au_8_Ge^−^ and Au_8_Sn^−^ clusters, we believe that isomer 4 should be a primary structure of the Au_8_Ge^−^ cluster. To further verify those structures analyzed by our method, the distributions of conformational populations were calculated at the MP2/ Def2-TZVPPD level of theory (using Gaussian09 software package, revision D.02, Gaussian, Inc.), and the results are summarized in Table 2. The conformational populations depending on temperature can be found in Figure S2. The results show those primary structures of the Au_8_M^−^ (M = Si, Ge, Sn) system obtained by our method hold a high percentage in the range of 1 to 500 K. For the structure analysis, the contrast of multi-dimensional characters, including energy, PES, and infrared spectra, can more accurately distinguish the different isomer configurations and reduce the uncertainty of the structure assignment. Through structure searching, the global minimum structure of M_N+1_ usually can be found from one of the low-lying isomers of M_N_ using our previous calculations^32^,^33^,^34^. Therefore, we have speculated that some low-lying isomers of the Au_8_Ge^−^ and Au_8_Sn^−^ systems may coexist under certain experimental conditions. Two possible evolution routes are presented in Fig. 3 for the Au_8_Ge^−^ and Au_8_Sn^−^ systems. The structure evolution routes provide us with another method to analyze the global minimum structure.

## Discussion

In this study, we have presented a three-dimensional structural assignment method based on energies and DOS spectra to distinguish different isomers. The method first involves exploring the PES using an optimization algorithm and obtaining sufficient isomers. Secondly, the DOS spectra of the low energy structures were simulated based on a generalized Koopmann’s theorem. The three-dimensional characteristics of cluster structure can be seen as the fingerprint of different isomers. This method will be more effective to determine which structures exist under real conditions. Applying the method to the Au_8_M^−^ (M = Si, Ge, Sn) systems, we found that the primary structures of Au_8_Ge^−^ and Au_8_Sn^−^ cluster are different from previous studies. The three-dimensional structure assignment method is simple and effective for different types of clusters to distinguish their structures.

## Methods

The basin hopping (BH) algorithm combined with density functional theory has been used to search the potential energy surface (PES). Generalized gradient approximation in the Perdue-Burke-Ernzerhof (PBE) functional and the double-numerical polarized (DNP) basis set with effective core potentials (ECPs), implemented in the DMol^3^ code^35^, were chosen for structure optimization of the Au_8_M^−^ (M = Si, Ge, Sn) system. More than 200 possible structures were produced by the BH method for the Au_8_M^−^ (M = Si, Ge, Sn) systems. The top 5 isomers were chosen based on their relative energies. The top 5 isomers were re-optimized using the functional PBEPBE and a scalar relativistic effective core potential Stuttgart/Dresden (SDD) basis set for all of elements in the Gaussian 09 software package (revision D.02, Gaussian, Inc.). The DOS spectra for all candidate isomers were calculated using PBE0 functional and CRENBL basis set for Si, Ge, and Sn, CRENBL basis set for Au with spin-orbit effects included in the NWChem software package^30^.

The DOS spectra were calculated using the following steps: (i) calculate the first vertical detachment energies (VDEs) of anion clusters, which was defined as the energy difference between the optimized anion isomer and the neutral at the corresponding anion geometry, (ii) add the binding energies of deeper orbitals of the anion cluster to the first VDEs to approximate the higher binding energy detachment features, and (iii) fit each peak with a 35-meV-wide Gaussian curve. Each peak can be seen as a molecular orbital. Therefore, we can obtain the approximate electronic structures of the anion cluster using this method. Because each anion cluster has a unique electronic structure, we can easily distinguish the difference of each isomer by their DOS spectra. This method is very effective for anion clusters, and we used this method to study the pure Au or doped-Au anion clusters in previous studies^33^,^34^,^36^,^37^,^38^,^39^,^40^.

## Additional Information

**How to cite this article**: Liu, Y.-R. *et al*. Three-Dimensional Assignment of the Structures of Atomic Clusters: an Example of Au_8_M (M=Si, Ge, Sn) Anion Clusters. *Sci. Rep*. **5**, 17738; doi: 10.1038/srep17738 (2015).

## Supplementary Material

Supplementary Information

## Figures and Tables

**Table 1:** Relative Energies of Five Low-Lying Isomers of Au_8_M^−^ (M = Si, Ge, Sn) at the PBE0/CRENBL(SO) Level of Theory using NWChem Software Package (CRENBL basis set for Au with spin-orbit effects included and CRENBL basis set for Si, Ge, and Sn), as well as PBEPBE/Def2-TZVPPD//PBEPBE/SDD,PBE1PBE/Def2-TZVPPD//PBEPBE/SDD, B3LYP/Def2-TZVPPD//PBEPBE/SDD, and MP2/Def2-TZVPPD//PBEPBE/SDD Levels of Theory using the Gaussian 09 Software Package (revision D.02, Gaussian, Inc.)^a^.

Relative energies (eV)							VDE (eV)	
isomer		NWChem	PBEPBE	PBE1PBE	B3LYP	MP2	NWChem	exptl^b^
Au_8_Si^−^	1	**0.000**	**0.000**	**0.000**	**0.000**	**0.000**	3.18	3.23 (3)
	2	0.122	0.150	0.211	0.186	0.370	2.69	
	3	0.169	0.156	0.261	0.268	0.410	2.73	
	4	0.214	0.212	0.257	0.268	0.301	3.12	
	5	0.291	0.306	0.399	0.415	0.246	2.59	
Au_8_Ge^−^	1	**0.000**	**0.000**	0.018	0.040	0.190	2.69	2.73 (4)
	2	0.031	0.015	**0.000**	**0.000**	0.192	2.61	
	3	0.118	0.183	0.112	0.198	0.010	3.15	
	4	0.134	0.155	0.145	0.204	**0.000**	2.52	
	5	0.183	0.193	0.179	0.191	0.353	2.38	
Au_8_Sn^−^	1	**0.000**	**0.000**	**0.000**	**0.000**	0.096	2.62	2.74 (4)
	2	0.194	0.208	0.173	0.221	**0.000**	2.48	
	3	0.194	0.185	0.264	0.193	0.447	2.79	
	4	0.317	0.328	0.250	0.313	0.098	3.10	
	5	0.325	0.298	0.210	0.209	0.327	3.24	

^a^Isomers are ranked according to their relative energies at five different levels of theory. The VDEs are computed at PBE0/CRENBL level using the NWChem software package and compared to the experimental values. Energies of the lowest-energy isomers are highlighted in bold.

^b^Reference 31.

**Table 2:** MP2-Calculated Relative Energies (ev) and Conformational Population (%) for Five Low-Lying Isomers of Au_8_M^−^ (M = Si, Ge, Sn) Systems.

isomer		∆E_def2-TZVPPD_^a^	%^b^
Au_8_Si^−^	1	0.000	99.987
	2	0.370	0.015
	3	0.410	0.000
	4	0.301	0.003
	5	0.246	0.010
Au_8_Ge^−^	1	0.190	0.009
	2	0.192	0.010
	3	0.010	21.156
	4	0.000	78.825
	5	0.353	0.000
Au_8_Sn^−^	1	0.096	0.735
	2	0.000	97.832
	3	0.477	0.000
	4	0.098	1.433
	5	0.327	0.000

^a^Relative to the lowest energy at MP2/Def2-TZVPPD theory level/basis set.

^b^Calculated using free energy values from Gaussian09 according to ∆G = −*RT*ln*K*. T = 298.15 K.
