# Supplementary material for: Three-Dimensional Assignment of the Structures of Atomic Clusters: an Example of Au8M (M=Si, Ge, Sn) Anion Clusters
Source: Sci Rep. 2015 Dec 3;5:17738. doi: 10.1038/srep17738 (PMC4668548; doi:10.1038/srep17738)
Supplement: Supplementary Information [file srep17738-s1.doc]

**Supporting Information**

**Three-Dimensional Assignment of the Structures of Atomic Clusters: an Example of Au_8_M (M=Si, Ge, Sn) Anion Clusters****

*Yi-Rong Liu, Teng Huang, Yan-Bo Gai,Yang Zhang, Ya-Juan Feng, Wei Huang**


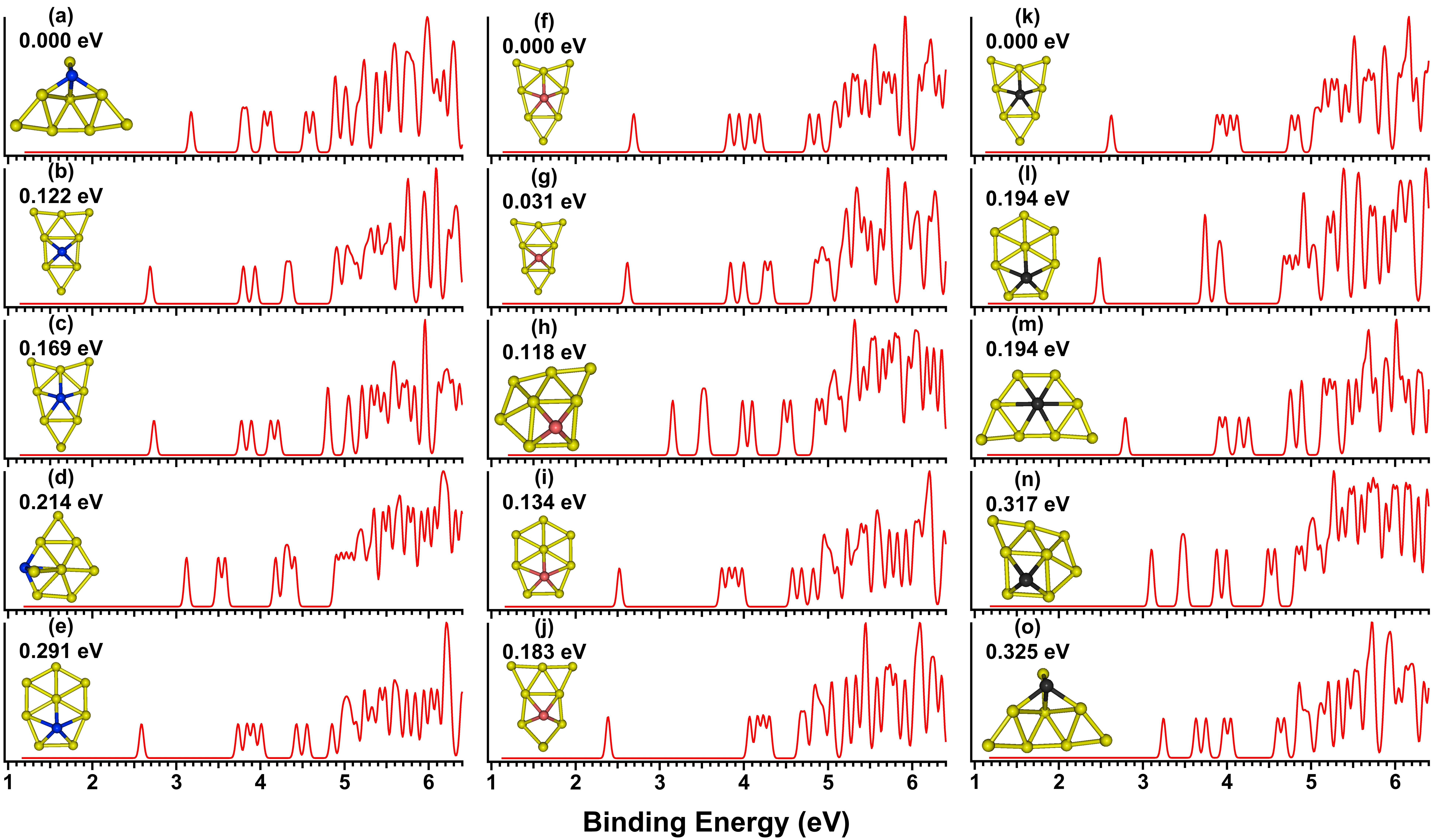


**Figure. S1.** The simulated PES spectra of the top-five lowest-lying isomers of Au_8_Si‾ (left, a-e), Au_8_Ge‾ (middle, f-j), and Au_8_Sn‾ (right, k-o). For each species, the isomers are numbered from the top to bottom (i.e. a-e) as isomer 1 to 5 in accordance with those in Table 1. The relative energies of five low-lying isomers were obtained at the PBE0/CRENBL level using NWChem software package (CRENBL basis set for Au with spin-orbit effects included and CRENBL basis set for Si, Ge, and Sn). The insets show the corresponding structures. The dopant atoms are shown in color (Si in blue, Ge in red, and Sn in black).

**
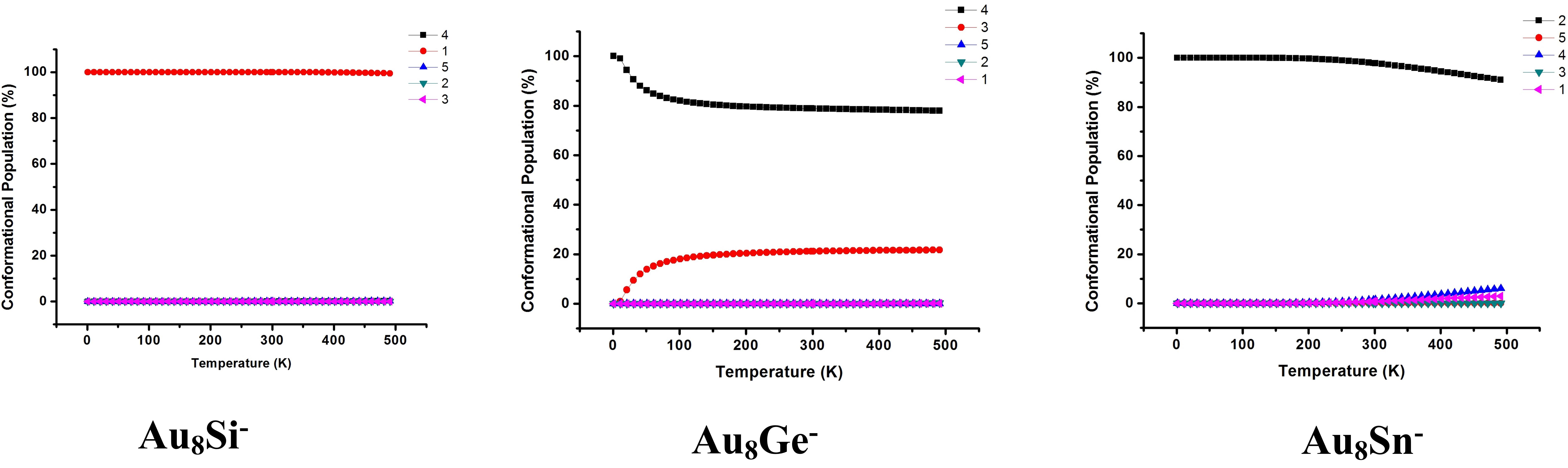
**

**Figure. S2.** The conformational populations depend on temperature for Au_8_M‾ (M=Si, Ge, Sn) systems. The temperature changes from 1K to 500K.

**Data S1:** The coordinates of low-lying isomers of Au_8_M‾ (M=Si, Ge, Sn)

**Au_8_Si‾**

Isomer 1

Au 1.3530131728 0.3937507589 -3.7680248656

Au 1.3530131728 0.3937507589 3.7680248656

Au -0.8186024100 -1.1293885446 0.0000000000

Au 1.5493608399 -0.7208906102 -1.3333655972

Au -0.6002686208 1.0471335408 -2.0218426047

Au -0.6002686208 1.0471335408 2.0218426047

Au 1.5493608399 -0.7208906102 1.3333655972

Au -3.4637765897 -0.5350566117 0.0000000000

Si -1.8715419611 1.3113802577 0.0000000000

Isomer 2

Au 0.2186394584 -0.4158826665 -1.5964662981

Au -0.2346023746 -2.8996568581 -2.6282499194

Au 0.0335998761 2.2572133704 -1.4241067906

Au -0.0230670708 -2.6423950287 0.0000000000

Au -0.3473828352 4.5544539398 0.0000000000

Au -0.2346023746 -2.8996568581 2.6282499194

Au 0.0335998761 2.2572133704 1.4241067906

Au 0.2186394584 -0.4158826665 1.5964662981

Si 1.6382877007 0.9277736723 0.0000000000

Isomer 3

Au 0.1395844428 -0.3691742965 -2.0431313298

Au 0.0725020812 -3.0061747827 2.5639683160

Au 0.0258254413 2.1916668289 -1.4169607589

Au 0.0725020812 -3.0061747827 -2.5639683160

Au 0.1395844428 -0.3691742965 2.0431313298

Au 0.0258254413 2.1916668289 1.4169607589

Au -0.2434199945 -2.2042643991 0.0000000000

Au -0.0117290249 4.4983603158 0.0000000000

Si -1.1755359681 0.3775352250 0.0000000000

Isomer 4

Au 0.0096248180 0.6112015809 0.5772682648

Au 0.3475612456 -3.9247683675 -0.4803818087

Au 1.4799333380 -1.4693753879 -0.6535922219

Au -1.1692554659 -1.8909267460 0.2865772092

Au -2.7644236955 0.1889631370 0.7223158164

Au 2.5078831320 1.3802308163 1.5701989575

Au -1.7111271331 2.4081319394 -0.5602593145

Au 0.8833631428 2.5638310668 -1.3496157260

Si 2.4124847774 0.7674166753 -0.7721281814

Isomer 5

Au 2.7550269859 -1.5517320452 0.0773788030

Au 0.6048606827 -3.2722242707 0.2116684278

Au -1.8888354896 2.8891017461 0.1285039326

Au 0.1551425756 -0.6403293870 -0.2264876486

Au 0.8750515592 3.3546810650 0.2144207723

Au -2.4561221339 0.2765999396 -0.1693029923

Au 2.3085751212 1.0966006438 -0.0842766272

Au -1.9938965183 -2.3731482003 0.0013881196

Si -0.2116944886 1.6287434601 -1.2458333537

**Au_8_Ge‾**

Isomer 1

Au 1.4295217185 -2.1744499383 0.0494610892

Au -2.0769145854 0.3760366980 0.1403004438

Au -1.4277255886 -2.1757095822 0.0629473938

Au 2.5555175701 3.0205011269 0.1290856701

Au 2.0789920059 0.3774994200 0.1164120275

Au 0.0016050988 -4.4725743995 0.0447487759

Au -2.5597508116 3.0186458319 0.1028295872

Au -0.0007862898 2.1979657423 -0.2053945813

Ge -0.0060751811 -0.3657480003 -1.2352779513

Isomer 2

Au -1.3797708424 -2.2499737631 -0.0200935676

Au 1.6606022470 0.4536583411 0.1484621906

Au 1.4659972021 -2.2075285983 -0.0324222898

Au -2.6690324780 2.9029803030 -0.2196542813

Au -1.6727025346 0.4009578425 0.1685029053

Au 0.0728046024 -4.5338543765 -0.3521803155

Au 2.5759339825 2.9855099571 -0.2496386212

Au -0.0399892682 2.5956768062 -0.0058473199

Ge 0.0256952876 -0.8543771041 1.6280255104

Isomer 3

Au -2.6283722068 -1.5697357034 0.4912177845

Au 2.3819297191 -2.3130158215 0.6244438340

Au -1.2867977125 3.4904450115 -0.0917860006

Au 0.9409098707 2.0808437575 0.1728386924

Au -0.2618058986 -3.0185130693 0.2202892268

Au -1.5668627090 0.8285725413 -0.5949585569

Au 2.9847995625 0.4369623801 0.7196199909

Au 0.7979747855 -0.5538034615 -0.6527739292

Ge -1.3442609655 -1.5759061215 -1.6605296508

Isomer 4

Au -0.6803194550 -3.3275045142 -0.1782389914

Au -0.7310023927 3.3605110129 -0.3178754978

Au -0.1403946805 -0.7132424525 0.1925333718

Au 2.0132564903 2.8046742682 -0.3133071761

Au 2.4700912473 0.1757825537 0.0764713475

Au -2.7673628977 -1.5047443461 -0.0071191661

Au 1.9573149761 -2.4768081876 -0.0461751190

Au -2.1947634286 1.1343168719 0.1008198323

Ge 0.3357390546 1.5951875022 1.2131686862

Isomer 5

Au -0.0104252121 -4.1443622819 0.4450554920

Au 1.4673538974 0.3722120224 0.0422805487

Au 1.7580318284 -2.2193848942 -0.5388270333

Au -2.6665298106 2.8030385055 -0.0215039128

Au -1.4679582128 0.3761758807 0.0165672377

Au -1.7551652431 -2.2142561741 -0.5707792605

Au 2.6724688971 2.7959783110 0.0220456598

Au 0.0012663577 2.6652793155 0.2047189284

Ge -0.0130011711 -1.5325004035 1.1637752167

**Au_8_Sn‾**

Isomer 1

Au 0.1804247137 0.3806709672 2.1176791980

Au 0.1557949282 3.0266481396 2.5701618435

Au 0.1804247137 0.3806709672 -2.1176791980

Au 0.1376005766 -4.4534420440 0.0000000000

Au 0.1178978854 -2.1735041885 -1.4527095305

Au -0.1167323508 2.2100711570 0.0000000000

Au 0.1557949282 3.0266481396 -2.5701618435

Au 0.1178978854 -2.1735041885 1.4527095305

Sn -1.4763780875 -0.3526613540 0.0000000000

Isomer 2

Au 0.2372896489 -3.4619419855 0.0000000000

Au 0.1012713970 -2.0922522383 -2.4054777233

Au 0.0038229304 0.6045984641 -2.3752695619

Au -0.3095793061 -0.8133812663 0.0000000000

Au 0.1012713970 -2.0922522383 2.4054777233

Au 0.3987760157 3.0965160500 -1.4087575432

Au 0.3987760157 3.0965160500 1.4087575432

Au 0.0038229304 0.6045984641 2.3752695619

Sn -1.4908583596 1.6732606611 0.0000000000

Isomer 3

Au 1.8679545463 -0.0329957709 3.9629393365

Au 1.6660261169 0.2103203473 1.2991449754

Au 1.6660261169 0.2103203473 -1.2991449754

Au 1.8679545463 -0.0329957709 -3.9629393365

Au -0.6121331590 -0.0186641143 -2.9211827396

Au -2.7340183736 0.1776402314 1.3568807122

Au -2.7340183736 0.1776402314 -1.3568807122

Au -0.6121331590 -0.0186641143 2.9211827396

Sn -0.5798530349 -1.0508344193 0.0000000000

Isomer 4

Au 2.8149644177 -0.6440406452 1.0067522193

Au 1.0001154628 -2.6628701751 0.3138875386

Au -0.5006553222 -0.4985848603 -0.7905813308

Au 1.3637548363 1.4593802967 -0.2781387338

Au 0.2822316543 3.9088444773 0.2473611047

Au -1.7512400379 -2.6933615609 0.1627242762

Au -1.4807398375 1.9406077846 0.0906630073

Au -3.0611302263 -0.2141008044 0.2389726146

Sn 2.1179408534 -0.9488350984 -1.5775913866

Isomer 5

Au -3.3530371777 0.9674040215 0.0000000000

Au 1.6761276757 0.6490181999 -1.3332859766

Au -0.5469537995 -0.9252846299 2.1715301662

Au 1.5185287866 -0.3123376694 3.8261535188

Au 1.6761276757 0.6490181999 1.3332859766

Au -0.6725535836 1.1035536605 0.0000000000

Au 1.5185287866 -0.3123376694 -3.8261535188

Au -0.5469537995 -0.9252846299 -2.1715301662

Sn -2.0233314936 -1.4240586033 0.0000000000

**Data S2:** Harmonic frequencies (in cm^-1^) and IR intensities of the low-lying isomers of Au8M- (M=Si, Ge, Sn)

**Au8Si^-^**

**Isomer 1**

# Frequency Infrared

1 17.3095 0.0030

2 20.8286 0.0303

3 34.8452 0.0301

4 37.0761 0.0556

5 44.1565 0.0674

6 52.0600 0.0075

7 55.8370 0.3077

8 61.8830 0.3441

9 62.7047 0.1939

10 65.4080 0.0644

11 77.3210 0.0842

12 89.9782 0.0152

13 96.1397 0.2291

14 105.8466 0.0009

15 119.9108 0.0069

16 142.1923 10.6613

17 142.4226 1.9815

18 175.3314 0.2408

19 252.1401 5.8636

20 313.2618 3.7606

21 355.1633 0.1918

**Isomer 2**

**# Frequency Infrared**

1 15.0943 0.0003

2 16.5129 0.0853

3 29.7049 0.0585

4 32.8501 0.1941

5 39.3568 0.0012

6 40.0217 0.0024

7 47.1205 0.0001

8 60.9277 0.5342

9 62.6839 0.0321

10 74.1208 0.0000

11 77.5350 0.6455

12 97.4633 0.0038

13 98.4764 0.5656

14 109.7431 2.8171

15 133.0410 7.9323

16 137.9167 1.1913

17 164.8610 0.2528

18 183.1864 4.4900

19 226.5620 1.1219

20 260.1292 0.0761

21 306.5608 4.8397

**Isomer 3**

**# Frequency Infrared**

1 17.2307 0.0147

2 20.1817 0.0332

3 30.4216 0.0389

4 32.3467 0.0028

5 38.5234 0.0170

6 42.9409 0.0019

7 53.4605 0.0008

8 57.1966 0.1581

9 58.4104 0.0118

10 74.5635 0.6441

11 80.5251 0.0727

12 95.3235 0.0442

13 101.8678 0.3992

14 106.8656 0.0132

15 136.6399 11.4775

16 146.5570 2.1917

17 178.2402 0.0741

18 181.0730 1.5589

19 203.5218 1.6191

20 250.7210 1.2902

21 270.3050 0.6003

**Isomer 4**

**# Frequency Infrared**

1 14.8397 0.0038

2 19.0503 0.0219

3 30.4511 0.0140

4 31.7426 0.1137

5 35.4123 0.1145

6 42.3954 0.2023

7 53.3327 0.4704

8 55.8783 0.1839

9 57.5621 0.1748

10 69.3038 0.4381

11 73.8681 0.2388

12 87.7630 0.1308

13 95.0129 0.2700

14 109.6784 0.2429

15 120.0580 0.5112

16 131.6911 0.8647

17 137.7886 0.6845

18 171.5973 3.8857

19 245.4432 9.4233

20 294.4975 2.9551

21 339.2419 0.4871

**Isomer 5**

**# Frequency Infrared**

1 11.2184 0.0396

2 20.6328 0.0554

3 21.8171 0.1123

4 27.2439 0.0153

5 29.4160 0.0252

6 31.5220 0.0072

7 41.5040 0.0014

8 59.8285 0.0000

9 68.0275 0.0847

10 78.9010 0.3740

11 82.4802 0.0535

12 88.6776 0.0390

13 103.6480 0.0322

14 106.4812 0.0423

15 135.9631 0.4999

16 140.5963 1.7642

17 150.5928 0.1438

18 161.6462 0.5444

19 199.1893 4.7981

20 245.4862 0.1866

21 298.4754 0.8721

**Au8Ge^-^**

**Isomer 1**

**# Frequency Infrared**

1 16.8351 0.0326

2 20.4072 0.0334

3 32.0528 0.0087

4 38.8561 0.0195

5 39.0948 0.0129

6 48.0951 0.0156

7 55.5984 0.0439

8 56.7493 0.0839

9 59.4330 0.0219

10 73.0733 0.7932

11 76.5274 0.0853

12 88.7010 0.0070

13 101.7033 0.0131

14 102.6833 0.4127

15 135.8410 9.3251

16 138.9227 0.9166

17 152.5407 4.0394

18 163.8049 0.6049

19 172.8815 3.1509

20 191.0742 0.7738

21 193.4673 0.0569

**Isomer 2**

**# Frequency Infrared**

1 14.7411 0.1189

2 15.2830 0.0065

3 24.0984 0.0154

4 32.6177 0.1764

5 39.7870 0.0037

6 40.3806 0.0020

7 48.9436 0.0003

8 59.9450 0.5367

9 62.6803 0.0142

10 73.2141 0.6725

11 75.2748 0.0588

12 93.6964 0.0524

13 98.2564 0.5639

14 109.9443 2.5953

15 129.7995 0.7303

16 132.7879 7.7079

17 148.9055 1.2369

18 164.1336 0.5668

19 173.8460 3.3901

20 193.1445 1.3378

21 208.4349 2.7918

**Isomer 3**

**# Frequency Infrared**

1 16.3121 0.0029

2 24.9108 0.0232

3 27.1509 0.0188

4 34.9968 0.0914

5 37.8333 0.1088

6 50.3519 0.0514

7 54.2099 0.1113

8 57.6210 0.1106

9 63.9154 0.0477

10 71.8404 0.0037

11 76.9867 0.1455

12 89.6706 0.3206

13 92.0220 0.0571

14 111.7415 0.0443

15 114.2575 0.1892

16 124.9532 0.8845

17 145.8245 0.3194

18 154.4437 2.3206

19 169.3504 2.6648

20 176.7906 1.0417

21 219.0983 1.2466

**Isomer 4**

**# Frequency Infrared**

1 13.0668 0.0264

2 20.6929 0.0265

3 26.0210 0.0531

4 30.7957 0.0268

5 33.2100 0.0256

6 40.1790 0.0618

7 45.3851 0.0061

8 61.6040 0.0209

9 70.7576 0.1526

10 78.0555 0.2405

11 79.1790 0.0212

12 84.6314 0.0161

13 98.8877 0.0012

14 101.7164 0.0584

15 126.3389 0.0450

16 131.3273 1.6198

17 147.8044 0.0431

18 149.7005 2.3459

19 164.2108 1.2214

20 181.8069 0.5773

21 192.9342 0.6260

**Isomer 5**

**# Frequency Infrared**

1 13.1933 0.0742

2 13.5665 0.0005

3 23.0944 0.0489

4 35.1599 0.0198

5 36.2445 0.0300

6 40.9537 0.0196

7 46.0260 0.0770

8 49.3628 0.0252

9 61.2785 0.0032

10 65.6353 0.7159

11 78.1269 0.5309

12 90.7756 0.1308

13 94.7418 0.8843

14 107.2677 0.0328

15 117.3282 6.6127

16 134.7176 0.6852

17 148.2563 8.0346

18 171.0343 4.0207

19 171.6261 0.7968

20 190.5540 0.0893

21 194.7625 2.6061

**Au8Sn^-^**

**Isomer 1**

**# Frequency Infrared**

1 16.6585 0.0502

2 20.1814 0.0329

3 31.2764 0.0107

4 38.8128 0.0151

5 39.3935 0.0000

6 51.3561 0.0446

7 54.2139 0.0196

8 55.5943 0.1934

9 60.7346 0.0223

10 67.4192 0.0720

11 70.7832 0.7609

12 82.2520 0.1003

13 96.4985 0.0461

14 103.4799 0.4178

15 118.7787 0.0385

16 125.5669 0.1858

17 136.7678 12.3967

18 146.2758 3.2512

19 157.4337 3.5921

20 182.6710 0.2974

21 183.9682 0.3888

**Isomer 2**

**# Frequency Infrared**

1 15.2608 0.0204

2 21.3857 0.0186

3 25.7394 0.0929

4 31.3342 0.0675

5 34.4891 0.0104

6 42.7506 0.0468

7 48.8096 0.0394

8 60.6099 0.0072

9 69.4818 0.0983

10 72.5387 0.0578

11 75.5061 0.2304

12 79.9277 0.0961

13 92.4504 0.0325

14 97.8993 0.1682

15 112.2287 0.3326

16 117.8865 1.2849

17 139.2935 1.3178

18 143.9966 0.3543

19 160.8672 0.7723

20 161.1426 1.1121

21 166.3854 1.1833

**Isomer 3**

**# Frequency Infrared**

1 11.7161 0.2214

2 13.1408 0.3255

3 14.6618 0.0216

4 19.1669 0.0220

5 30.6034 0.0444

6 33.5916 0.0316

7 36.6456 0.0022

8 51.6512 0.1023

9 59.7236 0.0957

10 75.5006 0.0612

11 78.1739 0.0719

12 81.2831 0.4551

13 85.6700 0.9431

14 99.6590 0.7538

15 111.8743 0.6896

16 120.9917 1.5349

17 130.2849 7.0408

18 144.9711 3.9946

19 165.1127 3.4133

20 166.7308 2.0144

21 195.4968 0.0924

**Isomer 4**

**# Frequency Infrared**

1 15.3560 0.0008

2 23.0632 0.0261

3 27.4254 0.0213

4 34.6835 0.1306

5 36.0097 0.1111

6 47.2313 0.1107

7 51.8228 0.0324

8 55.5217 0.0552

9 60.6362 0.0617

10 66.7734 0.0101

11 74.7810 0.0581

12 85.5212 0.1369

13 89.5011 0.3049

14 103.6928 0.5065

15 109.9435 0.1810

16 115.4280 0.7409

17 128.5970 0.1545

18 141.1088 1.6208

19 153.1226 1.3074

20 168.9181 3.5545

21 182.6378 1.4375

**Isomer 5**

**# Frequency Infrared**

1 17.2389 0.0109

2 17.7472 0.0004

3 31.5638 0.0083

4 37.2319 0.1296

5 37.8581 0.0452

6 46.1031 0.0003

7 46.3044 0.0911

8 58.0742 0.0869

9 61.6592 0.1817

10 62.4914 0.3690

11 75.4032 0.1239

12 84.9303 0.1414

13 88.1237 0.0723

14 107.1519 0.2044

15 107.4183 0.2649

16 134.2134 0.9013

17 137.1202 8.6260

18 150.1694 1.7462

19 164.2920 4.0272

20 172.8669 2.8933

21 180.0719 0.0044
